# Tokophobia or post-traumatic stress disorder ? about a tunisian case

**DOI:** 10.1192/j.eurpsy.2021.1207

**Published:** 2021-08-13

**Authors:** E. Cherif, L.S. Meddouri, A. Hajri, A. Maamri, H. Zalila

**Affiliations:** 1 Psychiatry, Outpatient Service, Razi Hospital, Manouba, Tunisia; 2 Outpatients Ward Of Psychiatry, Razi hospital, Manouba, Tunisia

## Abstract

**Introduction:**

Pregnancy and delivery are considered to be an important transition stage in a woman’s life. Although this experience is emotionally rich, it varies from one person to another and each woman goes through it in her own way.

**Objectives:**

discuss the psychiatric outcomes after a childbirth with somatic complications.

**Methods:**

case report

**Results:**

Mrs X is a 32 years old woman, she has no particular history of illness until she gave birth to her son. He is now three and a half years old and he is an outpatient at the child and teen psychiatry department in a Tunisian hospital. After her delivery, Mrs X had several physical and psychological complications. She was hospitalized in the cardiology department for cardiomyopathy of Meadows for three weeks among it one week in the medical reanimation ward because she needed respiratory assistance. Furthermore, she suffered of left femoral head’s necrosis for which she was operated, and a total hip prosthesis replacement was done. Psychologically, Mrs X. presented a postpartum depression which resolved in its own after 9 months. Ever Since the childbirth, the patient presents symptoms concording with post-traumatic stress disorder and symptoms that may be linked to a specific phobia (fear of birthchild or tokophobia).

**Conclusions:**

In addition to the usual health care provided to women during pregnancy and after childbirth, looking for mental health disturbances and eventually referring them for psychiatric assessment is important specially for women who have experienced traumatic events during the pregnancy or the delivery

**Conflict of interest:**

No significant relationships.

